# On the Analysis of the Blood and Urine in Health and Disease, and the Treatment of Urinary Diseases

**Published:** 1846-01

**Authors:** 


### On the Analysis of the Blood and Urine in Health and Disease, and the Treatment of Urinary Diseases. By *G. Owen Rees*, M.D. F.R.S. &c. London, Longman & Co. 1845.

In estimating the merits of a work which professes to treat upon any department of Organic Chemistry, it is well to bear in mind the different objects to which that science may be made subservient, and to endeavour to ascertain with what especial view it has been cultivated by the author before us, and what is the nature of the information which he designs to convey to others. Now we hold it to be sufficiently evident, both from the statements made in the prefaces and from the contents of the book itself, that it was never Dr. Rees' intention to push his analytical investigations to an extreme point of minuteness in detail, or to trace, except generally, the sources or characters of the various proximate principles which come under his notice. It appears, if possible, still farther from his purpose to enter upon the wide field of ultimate analysis, which we think would have been both unnecessary and unprofitable. He seems rather to have wished to exhibit to others the simplicity of the processes by which a considerable, if not a perfect, practical knowledge of the constitution of the important fluids of which he treats may be obtained, a knowledge which, in its application to individual cases, may assist diagnosis and guide treatment, and may, at least with some, be a starting point from which they may be tempted to press onward in the same course, and to take their parts in the more direct advancement of animal chemistry as concerned with vital actions either during health or under the influence of disease. In proof of what we are saying, in the preface to the former edition reprinted in the present, we find the author stating it to be his object "to exhibit a concise view of those plans of analysis which may be performed simply, usefully, and at small expence," and expressing the satisfaction which he will feel "if his volume should in any way increase the number of those who occupy their leisure hours with the study of animal analysis, as applied to disease;" while, in a new preface, he declares the present edition to be constructed on the plan of the first, with an addition on the treatment of urinary disease; but he asserts that he has avoided as much as possible entering into scientific details as foreign to the purpose of his work, which must not be regarded as an elaborate treatise on the blood and urine in all their relations, but simply as a work on proximate animal analysis. He states, at the outset, that considerable alterations have been rendered necessary by the advances made in animal chemistry since the first edition was published; but a careful collation of the two editions has enabled us to recognise but few of these, and of matter altogether new, yet admissible consistently with the plan of the work, there is scarcely so much as an interval of nine years might lead us to expect.

The instructions contained in the Introduction, and the cautions given in reference to the performance of Evaporation, Filtration, and Incineration, though not new, will be found necessary to beginners and useful to most readers; the recommendation of weighing by counterpoise where great accuracy is required, is certainly entitled to adoption, and not less important is the direction to note with the greatest care the result of every weighing performed during the analysis. Of the circular-wicked spirit lamp, Dr. Rees very justly remarks that it is a somewhat dangerous piece of apparatus in the hands of the beginner, he might have added that, where a long continued heat is necessary, it must also be an expensive one. He has omitted all mention of the use of a circular gas-burner, but this we have seen introduced as a source of heat in two laboratories, and we are disposed to believe that, in large towns at least, where gas is readily procurable, it might advantageously be resorted to as a cheap, very manageable, and, for operations on a small scale, very sufficient heating power. The necessity of being provided with absolutely pure re-agents, is very properly insisted upon, and the common impurities existing in some of them are briefly pointed out; and we come next to two lists, one of chemicals and one of instruments of analysis, very encouraging from their brevity and attractive from their precision to the student about to put in practice the methods of analysis to be taught in the subsequent pages. We are told that these instruments will render our laboratory pretty complete, which amounts to saying that the list given is pretty complete. Now we protest decidedly against such a list being pretty complete, or nearly complete, we hold that it should be absolutely complete, and that the author should afterwards employ no article of *materiel* not previously set down, as the counsel in a prosecution can summon no witness not named beforehand.

The adoption of any less stringent rule than this is sure to result in misconception and disappointment; accordingly we shall see that the beginner, who, trusting to his list, has supplied himself with almost all things needful, will find himself at a loss for the stoppered bottle and bits of lead required in the very first analysis. It is true that these are mere trifles, and for the most part easily procurable, but the time of discovering the deficiency may prove a very inconvenient one for remedying it. Farther on, it is startling to perceive that, for determining the quantity of sugar in diabetic urine, we are to make use of so important an addition to our stock as a mercurial trough with a graduated receiver. Our own wants, too, must teach us to provide both an instrument for determining the specific gravity of urine and of saline solutions, and one also for securing in our alcohol, and spirits of wine, the various degrees of strength at which for different purposes we are directed to employ them. A thermometer too is an omitted article, though we meet subsequently with several occasions for its use.

An account of the visible structure of the blood as it is made known to us by the microscope, precedes a description of its analysis. The presence of fibrinous corpuscles, as well of blood-corpuscles, in the compound liquor sanguinis is spoken of, and the separation of this last into crassamentum and serum is described. This spontaneous coagulation, we may remark, as it occurs either in the whole mass of the blood or in a separated portion of the liquor sanguinis, is considered by Dr. Babington to indicate the extinction of its vitality. The constitution of the blood-corpuscles, as established in great measure by Dr. Rees' own researches, is detailed. We believe that the existence of a central nucleus, spoken of elsewhere by himself, though he does not here mention it, is generally admitted, as well as that of a membrane or envelope enclosing the fluid from which it derives its colour, and traversed by inward or outward currents according as the corpuscle be placed in a fluid of less or greater specific gravity than that of its own fluid contents.

The first problem in analysis is the determination of the proportion of Water, solid matters of Serum, Fibrin and Red Corpuscles in healthy Blood, and of this we shall give a condensed sketch, which will serve to show with what simplicity and distinctness the means of accomplishing a task of some difficulty and intricacy are set before us.

Three separate small quantities of the same blood are to be received in three distinct vessels, whereof one is a stoppered bottle and another a platinum capsule, the weight of each of these vessels being accurately known. For the determination of the proportion of fibrin, the blood in the bottle is to be agitated during coagulation with eight or ten small pieces of lead of known weight, which will cause the fibrin to attach itself to them. Now the balance will show us the weight of the bottle, lead, and blood together, and as those of the bottle and lead are known, the difference will be that of the blood operated upon. The bottle is now to be emptied and the fibrin carefully separated from the lead, collected, washed, dried and weighed. This gives us the quantity of fibrin in a certain known quantity of blood, and as the proportion is invariable for the same blood, we can calculate the quantity in any other weight of blood as in 1000 grains.

The weight of the blood in the capsule ascertained as before by subtracting that of the capsule alone from that of the capsule and blood together, is next to be noticed; and the capsule is to be placed over a water-bath, and the blood dried till it ceases to lose weight. By again weighing the capsule with its contents we now learn both the loss of weight, which is equal to the weight of water contained in the known quantity of blood, and the remaining weight, less the weight of the capsule, which shows the amount of solids in the same quantity of blood. Hence we deduce the quantity of water and solid matter respectively in any other quantity of the same blood as in 1000 grains.

We have before determined the weight of Fibrin in 1000 grains of blood, and by subtracting this from the weight last obtained we shall get the aggregate weight of the solids of the serum and of the solids of the corpuscles in 1000 grs. of blood.

We know then the weight of Fibrin
of Water
of solids of serum and of red corpuscles together.

By treating a portion of serum obtained after coagulation from the blood in the third vessel in exactly the same way as the blood in the platinum capsule was treated, we learn the relative proportions of water and solid matter contained in the serum; and, assuming that all the water of the blood exists in it in combination, so as to form serum, we can calculate the weight of solid matter of serum corresponding to the quantity of water found in the blood, and this weight, subtracted from the sum of the weights of the solids of serum and of red corpuscles, leaves us, of course, the weight of the solids of these latter.

It would be more correct to say that this process gives us the weight of dry globulin, it does not give the weight of the solids contained in the hæmatosine, unless that fluid be of greater density than the serum, in which case it gives the weight of the solids due to that difference in addition to that in the globulin: it gives a deficient weight if the hæmatosine alone be less dense.

The above process is new in the second edition, but as the blood cannot always be obtained before coagulation, the former method, which obtained the weight of fibrin from the clot, is given in a note.

Having given this specimen, we need not follow our author through the details of his analysis of serum, which is identical with that given in the former edition, we may remark in passing that we meet here, as elsewhere, with useful hints and cautions very valuable to a learner, and which could only have been suggested by one to whom the processes had been practically familiar. Of such a kind is the direction, p. 25, that on treating the dry residuum of the evaporation of serum with boiling distilled water, "care be taken that the heat he kept to 212° Fahr. at the moment of admixture, or otherwise the albumen is liable to assume a gelatinous form, which greatly interferes with the process." Again, the caution in the note at page 32 against using "too great a quantity of nitric acid for dissolving the earthy salts, because a great excess of nitrate of ammonia tends to hold the phosphate of silver in solution," is both useful and necessary.

The white matter of the blood-corpuscles called globulin, is said in its re-actions to resemble fibrin and albumen, being soluble in alkalis, and precipitable when so dissolved on neutralization with acids. The hæmatosine or red colouring matter of the blood-corpuscles is mentioned as yielding an ash rich in iron, and the author is of opinion that, if this metal be met with elsewhere, its presence is accidental and derived from the fluid of the corpuscles.

Dr. Rees, when asserting that should crystals appear in the alcohol extract of blood after the addition of nitric acid, they must be nitrate of urea, seems to have overlooked or disregarded the statement of Simon, who mentions and figures crystals of nitrate of soda as liable to occur under similar treatment of blood devoid of urea.

For detecting sugar in diabetic blood, Trommer's test is applied to the filtered fluid obtained by treating the dried serum with boiling distilled water. But the quantity of sugar present is determined by evaporating to dryness this filtered solution, and digesting the dried residuum in alcohol of sp. gr. 0·825, evaporating this filtered alcoholic solution again to dryness, treating with ether to remove fatty matter and some urea; again taking up the undissolved portion with alcohol, and leaving it to spontaneous evaporation, whereby is obtained a crop of mixed crystals principally composed of alkaline chloride and diabetic sugar, which are to be separated by careful manipulation. Dr. Rees has effected this separation by using alcohol of low specific gravity; for, when agitated in this, the chloride subsides before the crystals of sugar, and allows of the latter being poured off partly dissolved in the fluid. In this way he obtained 1·8 of diabetic sugar in the serum of a patient whose urine had a specific gravity of 1048, but he wishes this to be considered only as an approximative result.

In taking leave of the analysis of the blood, which terminates here, we may remark that the author has omitted all notice of the oxydes of Protein, which are stated by Mulder to exist in healthy, and, to a large extent, in inflamed blood; neither has he expressed any opinion on the question, whether it be the iron or the Protein of the blood by which the oxygen of the air is appropriated and distributed over the system; and we think that, in both these respects, he has exercised a sound discretion, since, in a work of a somewhat elementary character, we may well be content to forego the means of seeking for substances not mentioned in the analyses of Andral and Gavarret or of Simon; and the discussion of the physiological question is clearly beyond the intended scope of the treatise.

We propose to make some extracts from the portion of the work which treats of the urine, of urinary deposits, and of calculi. We shall next briefly advert to the contents of the Appendix, and shall then proceed to offer some remarks upon that entirely new portion of the book, which speaks of the treatment of urinary diseases.

We think that Dr. Rees would have done well, and not have departed from his general plan, if he had prefixed to his consideration of the urine some brief view of its normal properties, of the source of its acidity, of its average density, and of the variations to which it is in this respect liable, according as it passed shortly after drinking freely of fluids constituting the urina potus, or after the digestion of a principal meal, when its qualities may be referred to the fresh matters taken into the system, or again when it is excreted after a night's rest, and removes, in all probability, from the blood substances which are derived from a secondary assimilation.

Some notice of these differences of its origin and properties, we conceive, should have been taken, since inferences drawn from the chemical analysis in different cases, unless it be compared under similar circumstances, would have a tendency to mislead; and no calculation approaching to accuracy could be formed of the amount of solid egesta carried out by the kidneys, unless not only the quantity of urine, but its average density during the day, be approximatively ascertained.

The following is the method of analysis pursued for the determination of the quantity of urea, and a description of its property.

"Evaporate urine to the consistence of a strong syrup, and then add pure concentrated nitric acid, until the whole mass becomes more or less solid. The crystalline matter which is now produced consists of nitrate of urea. This must be washed from adherent impurities by ice-cold water, and then pressed between folds of bibulous paper to dry. These crystals are now to be dissolved in luke-warm distilled water, and neutralised with carbonate of barytes. This mixture is to be evaporated to dryness, and alcohol boiled on the dried mass. In this way the urea may be extracted from the barytic salt. It may be obtained in colourless crystals, by digesting the alcoholic solution with animal charcoal, then filtering, and allowing the urea to crystallise by spontaneous evaporation. The chemical properties of urea are as follows :—

"When heated on platinum foil it fuses; and if the heat be urged is decomposed, yielding fumes of carbonate of ammonia.

"It is very soluble in cold water, but more so in warm. It gives out a great degree of cold when dissolved in any considerable quantity.

"The concentrated solution in water will bear a heat of 212°, without decomposition; but in dilute solution it quickly decomposes at that temperature.

"Alcohol of specific gravity 0·816 dissolves a fifth of its weight of urea at 60° Fahrenheit; when boiling it dissolves nearly its own weight.

"It is slightly soluble in ether. The caustic alkalies decompose urea into carbonate of ammonia.

"The nitric and oxalic acids combine with urea, forming salts more or less insoluble. The crystallisation with nitric acid forms one of its best distinctive characters.

"Urea possesses neither an acid nor alkaline reaction; its crystalline form is that of a four-sided prism, exceedingly delicate, and silky in texture." P. 51.

This is all very good, and clear, so that we are at a loss to know why, when the same problem comes to be solved again at p. 64, a different process is recommended, viz. the use of oxalic acid and carbonate of lime, instead of nitric acid and carbonate of barytes.

There is a simplicity in uniformity much to be admired, whereas unnecessary difference is always embarrassing. In the search for urea in the blood at p. 40, in its qualitative and quantitative determination in the urine at p. 50 and 64 respectively, we think the beginner would be apt to recognize distinct problems rather than modifications of the same.

The separation of lithic acid, at p. 57, by hydrochloric acid, is simple and easy, as is its resolution in potassa, and re-precipitation by the same acid to free it from colouring matter; the detail of its chemical properties is deferred till we come to the analysis of urinary calculi.

The reactions of vesical mucus are given as its solubility in acetic and nitric acids, from which solutions it is precipitable by ferrocyanide of potassium ; its solubility in potass with the evolution of ammonia, and its non-solubility in sulphuric acid. Viscidity and transparency are, we believe, correctly spoken of as the properties of mucus, distinguishing it from pus, which is opaque and devoid of tenacity, at least when not modified by alkaline re-agents.

We are desired, in order to prove the presence of hydrochlorate of ammonia in the urine, to expose it for several days, by which means we produce a crystallization of various salts at the bottom of the vessel, consisting principally of hydrochlorate of ammonia, of chloride of sodium, and of ammoniaco-phosphate of soda mixed with earthy posphates. Now, as no one, we suppose, will contend that the formation of these salts is a result merely of spontaneous evaporation, without any decomposition and re-arrangement of the proximate principles of the urine, we think that such a method of testing is wholly inconclusive and unsatisfactory, unless it be shown, which has not been attempted in this case, that the decomposition and re-arrangement spoken of above do not, and can not, affect the composition of the particular salt or principle of which we are in search.

The presence of chlorides, phosphates, and sulphates, in combination with alkaline bases, is shown by testing the aqueous solution of the residue of the incineration of the solids of urine with nitrate of silver, and nitrate of baryta.

That the bases are alkaline, not earthy, is to be shown by adding a solution of carbonate of potassa to a portion of the same solution, as was done in testing the saline matters of the blood at p. 29; but, why the former process is not here referred to, and the determination of the alkaline bases, by means of tartaric acid, and the effect on the colour of flame, is not carried out as before, we are puzzled to find out. Tartaric acid in excess will act characteristically upon potass, whatever be its state of combination, and even where there is an excess of the strongest acid if ammonia be used in excess before the addition of tartaric acid; and this addition of both ammonia and tartaric acid would not interfere with the application of the test for soda to the evaporated residue of the fluid from which the potass had been thrown down.

At page 61, we have described the method of recognizing the earthy phosphates constituting the bulk of the remainder of the insoluble matters contained in the residue of the incineration of the solids of the urine. Now there is no reference here to the method described in page 31, for examining these phosphates as they exist in the blood; it is therefore a peculiarity not much to be commended, that we have here no allusion to the method of determining the acid in combination with the bases. In the former part, which treats of the blood, we have the phosphoric acid tested (after neutralizing the nitric solution of the phosphates with ammonia) by nitrate of silver, which does produce a characteristic yellow precipitate of phosphate of silver, not blackening however so readily as is stated; and the blackening not being distinctive of the presence of phosphoric acid if it did occur; and also by the effect of phosphoric acid in giving a green tinge to flame. In this part, as regards the bases, we are only told that, "in order to demonstrate the presence of the earthy phosphates, the acid solution must be neutralized with ammonia, when the peculiar gelatinous appearance of the precipitate will sufficiently characterize its nature." But at page 61, where the phosphates of the urine are examined, the determination of the acid is omitted, but we have the bases accurately determined by the precipitation of lime with oxalate of ammonia from the nearly neutral solution in nitric acid, and the subsequent precipitation of the ammoniaco-magnesian phosphate from the supernatant fluid by the addition of more ammonia; and we have thus the singular phenomenon of one and the same problem, viz. the determination of the nature of certain phosphates commenced on those derived from the blood, and completed on others, to be found in the urine. We are aware that the quantities operated on are minute, but we think that this will hardly excuse the incompleteness of either separate examination.

Dr. Prout's arrangement of the urinary deposits has been adopted; and the determination of their nature is simple and easy. Characteristic tests are given for the oxalate of lime deposit; we doubt whether this would be found insoluble in warm dilute nitric acid, if the dilute acid be as strong as that mentioned in the list of chemicals that is made, with one part of acid to nine of water. And, after all, the microscope, we believe, affords the best means of ascertaining the nature of this precipitate.

We wonder that, in treating of cystine, so characteristic a test as that proposed by Liebig should be omitted, and which consists in dissolving the calculus or gravel in a strong solution of caustic potass, and adding to the solution so much of a solution of acetate of lead, that all the oxide of lead is retained in solution. When this mixture is boiled there is formed a black precipitate of sulphuret of lead, which gives to the liquid the aspect of ink, Abundance of ammonia is also disengaged, and the alkaline fluid is found to contain, among other products, oxalic acid.

A list of the indications produced by different re-agents is given at page 77, and constitutes a kind of recapitulation of actions, for the most part mentioned before, but why it is inserted here, or why being inserted, other actions, and other agents are not included, we have no reason assigned, and are, therefore, content to remain in ignorance.

The fact that vegetable colouring matters exist occasionally in the urine, and that their nature may be detected by the green tint produced by potass in excess, and the re-production of the original colour by supersaturation with an acid, is one of practical importance. Dr. Rees expresses no opinion as to the fact of mercury having been detected in the urine, but states that he himself failed to discover any trace in the urine of a person suffering under mercurial salivation. Our own belief is, that it has never satisfactorily been proved to be present in this secretion, and that what was discovered by Cantu, must have gained accidental admission from without.

The quantity of sugar contained in diabetic urine is ascertained by fermentation, and collecting the carbonic acid evolved in a graduated jar over mercury, and allowing one grain of sugar for every cubic inch; this, no doubt is a sufficiently accurate method; our only objection to it here is, that it involves a slight departure from the simplicity and inexpensiveness of the means to the employment of which we are limited in this treatise.

We can not help here adducing another instance of Dr. Rees's extreme fondness for diversifying his processes, whereby, instead of bringing many individual cases under one rule, he makes many rules for one and the same case. Is sugar to be sought for? If we look for it in the blood, its presence, after the preliminary steps, is to be determined by Trommer's test. (p. 47). Do we seek it in the urine? Mr. Moore's test by Liquor Potassæ is recommended. (p. 80). Is its quantity to be determined? If in the blood, it is to be separated by means of alcohol from the evaporated residue of the watery extract. (p. 47.) If its quantity in the urine is to be ascertained; fermentation, as we have just mentioned, is to be employed. (p. 89).

Now we are far from asserting that altered circumstances, relating to the quantity of the priuciple sought for, or to its state of admixture do not render a modification or total change in the mode of operating necessary or advisable, but we think that an author creates perplexity when he wantonly adopts these variations, and that, in order to instruct to the best advantage, he should always explain the grounds of his preference.

The analysis of urinary calculi will not detain us long; they are divided into two classes, viz. those whose texture and composition are destroyed by a red heat, and those capable of resisting heat. In this section we have a repetition of processes with which in the former part of the work we had become familiar, as testing for lithic acid and lithate of ammonia; but oxalate of lime in a calculus receives a somewhat fuller consideration than it did as a sediment, and its decomposition by means of boiling in Carbonate of Potassa is spoken of, which perhaps affords the best means of satisfactorily proving its constitution. Cystine too meets with better treatment at this second mention than before, and we have now Liebig's process given, though not quite in full, the production of oxalic acid not being mentioned.

Dr. Rees has not adopted the term Uric Oxyde, or Urous Acid for the substance first discovered by Dr. Marcet and named by him Xanthic Acid; but seems somewhat to incline to the opinion that, it is a compound of uric acid and albumen.

In the Appendix we meet with a new formula for the more accurate determination of the proportions of lime and magnesia, of which we shall only say that we much doubt our need of it; and we next come to a repetition of the same process which has already been given three times, viz. at pages 61, 72, and 112.

We have, farther on, a more particular account of the manner of extracting the fatty matters of the blood and of their different characters; and then some interesting results of the examination of blood as derived from the researches of M. Lecanu and other experimenters.

The organic acids of urine are next spoken of, which we the more readily pass by in silence, because the subject has been discussed in a recent Article in this Review, when Dr. G. Bird's work was under examination.

Another note is then given on the quantitative estimation of fixed Alkaline Chlorides, Phosphates and Sulphates in the urine, describing a process precisely the same as that given at page 60, except that, as we must have variety, the phosphoric acid is here separated in combination with barytes instead of with silver: and the potass thrown down with chloride of platinum instead of tartaric acid, and we much doubt whether the alteration in either case be an improvement. Some remarks upon the characters of albuminous urine and the tests of this condition follow, but why these were not incorporated in the text it is difficult to discover. We think that a simple and certain test for the determination of the presence of albumen in the urine would most naturally have preceded that which is to show us how to determine its quantity. The fact of copaiba and cubebs, when administered internally, producing an appearance in the urine resembling that of albumen, when the tests of this last are applied, is interesting and important, and the means of distinction, viz. the non-subsidence of a precipitate in the former case after several days is a most useful and necessary matter of information. The pith of this note certainly deserves a more prominent situation in the body of the work.

A table follows indicating the frequency of the occurrence of albuminuria—this we consider valuable. The non-production of albuminous excretion from the kidneys, as a consequence of the action of mercury on the system, seems almost established by the examination, by Dr. Francis, of the urine of fifteen patients under the influence of the remedy.

It was scarcely necessary to tell us again here that, by boiling a portion of urine in which an unknown sediment is diffused, we might recognize the presence of lithates or phosphates, according as the liquid becomes clear or not. We have before, more than once, had occasion to learn that the lithates are soluble at a boiling temperature and the phosphates not so; the practical application of this fact would most naturally have been briefly indicated immediately after we had acquired a knowledge of it.

After a description of some deposits of matters accidental to urine, we have Dr. Henry's table for calculating the solid matters contained in Diabetic urine of different specific gravities. We cannot, in passing, avoid the remark, that all tables of the solid contents of urine go to establish the general fact that, as far as regards the matters contained in that fluid, solution never takes place without expansion; for otherwise, in a bulk of urine equal to that of 1000 grains of distilled water, we should always have exactly one grain of solid matter for each unit of increase in specific gravity. Now all the tables give much more than that amount; and this result can only be explained in the manner just pointed out.

Several authors have given us the law of this table so as to facilitate its use, but the rule deduced from it by Dr. Frampton, as published in the Medical Gazette, is the most easily remembered, and the most readily applied. It is this, multiply the number of degrees by which the density of the urine exceeds that of distilled water by 1·2, this will give you the solid contents of one fluidounce in grains, multiply this by the number of fluidounces, and you have the whole solid contents of the quantity of urine experimented upon.

A brief notice of Kiestein, which Dr. Rees considers a modified case in; and of chyloserous urine, in which he satisfied himself of the existence of chyle, closes the Appendix.

He who wishes to enter on the study of this branch of organic chemistry, with the assistance of Dr. Rees's book, would do well, we think, to begin his reading at the end, and to pursue his course in an inverted order till he comes to the beginning. By so doing he will have the advantage of becoming familiar with simple and easy processes before he comes back to the more complex and difficult with which the book sets out. This will be a great gain; not that it can make the arrangement altogether uniform or good. This Dr. Rees seems to guard against; accordingly, when he treats of blood, the quantitative precedes the qualitative analysis, when of urine, the order is reversed. Things so nearly a-kin as Urinary Deposits and Urinary Calculi are separated in his discussion of them to admit of the examination of albuminous and diabetic urine, the processes applied to which approach so nearly in character to those employed on healthy urine from which, therefore, they are divided. Deposits having been partially classified (some stray behind by themselves, being too bad we suppose, for any class) on one principle, namely, that of their colour and texture; the calculi are arranged on another, namely, that of their comportment under the influence of heat. The new matter contained in the present edition is rather brought in patches than properly incorporated. We are willing to believe that some things to which we have raised objections in this article, as the multiplicity of methods for accomplishing the same end, are a result rather of the embarras des richesses than of any other cause: others, and the greater number, are due to carelessness. It is matter of astonishment and regret to us that Dr. Rees has not been at more pains to set forth to better advantage the large store of accurate knowledge which he undoubtedly possesses. Had he merely published an account of the various experiments which he himself has made, such a work, like the present, would have been replete with interest and value, but why should not the best material be also moulded into the best form before it is set before the world.

We enter with no little interest upon the consideration of the treament of urinary diseases recommended by one so expert in chemical enquiries as our author. If the field in which he has more particularly laboured for the advancement of medical knowledge have been before broken and worked by others, his fellow-labourers have been comparatively few, not sufficing to exhaust the soil, from which we trust to gather much useful fruit. We gladly hail, therefore, the announcement of our author that, "on some points of practice, he has been led to form opinions materially affecting treatment, and not entirely devoid of novelty." We shall not follow Dr. Rees into an enquiry into the different constitutional states associated with deposits of lithic acid and the lithates, a subject upon which there is a nearer approach to unanimity than is common in the medical world. Our present concern is with treatment, as guided by certain chemical phenomena, and we extract a passage containing remarks, in the justice of which we entirely concur.

"When the red crystalline form of lithic acid is excreted, it has been very commonly supposed that the chief benefit we can derive is to be expected from the administration of alkaline remedies, which are known to correct the acid state of urine found in connection with this form of disease, and, moreover, to act as solvents on the deposit itself. It is a matter of fact that alkaline remedies will constantly cause the urine of patients suffering from the lithic acid deposit to become clear and transparent and that the symptoms of dysuria are generally relieved by the remedy; but I have seldom known this palliative treatment attended by any lasting benefit; and we must remember, that it should be our object to correct those general conditions of system on which the production of the unhealthy urine depends, rather than to afford a temporary benefit, by the use of means in themselves but ill calculated to effect the great object of all treatment." 125.

We have for some time past, like Dr. Rees, looked upon alkaline remedies as mere palliatives in the treatment of disease, tending, indeed, to the amelioration of some secondary inconvenience, as that arising from the presence of acid in the stomach, or from lithic acid in the urinary passages, but being so powerless against the original sources of mischief, that they must be taken in continually increasing quantities to keep the enemy at bay. This, we believe, is commonly the case with those who are in the habit of dosing themselves with carbonate of soda; and we once met with a young woman, who, from the nature of her employment, had more than ordinary facilities of obtaining chemical substances, with regard to whom, we were told, that a pound lasted only three weeks.

We are glad to see Dr. Rees, himself a chemist, resisting what he properly calls the dogmatism of the laboratory; and expressing his distrust in the ingenious doubling and mingling of atoms as a means of assisting us in the true nature of disease, or the plan best adapted for its cure.

Mild aperients, with vegetable tonics, regulated diet, moderate exercise, and the use of horse hair gloves, or of a flesh brush to excite the function of the skin are the expedients recommended as tending to give permanent relief. For some cases acid tonics are stated to be beneficial, and we think justly so.

In cases of severe dyspepsia, characterized by the deposition of lithates, in urine containing a small quantity of sugar, the prescription of opium with Ipecacuanha at night, and the use of Hydrochloric Acid in large doses is strongly recommended.

We have no particular fondness for apparent paradox, but think the remarks on some particular cases of alkaline urine depositing earthy phosphates, and treated with alkalies, too important to be omitted. There are cases in which the urine is supposed to be acid on its leaving the kidney, but, by its irritation of the internal surfaces of the ureters and bladder, to produce so copious a secretion from them of alkaline mucus as to cause its own acidity to be more than neutralized and the phosphates deposited. Dr. Rees thus states his reasoning and the successful issue of the treatment it suggested.

"It appeared to me that the use of alkaline remedies might be of advantage in these cases of alkaline urine, and, moreover, that if the alkali were administered in small doses so as only partially to neutralise the acid state of urine as secreted by the kidney, we might relieve the irritation of the mucous membranes, stop the excretion of alkaline matter, and have an acid urine excreted, the natural acidity having been only partially destroyed by our remedy. It may appear somewhat unaccountable to those who merely look to the chemical view of the matter that any one should expect to render alkaline urine acid by the administration of alkalies, but such was the treatment I adopted, and the result fully corroborated the correctness of the theory which suggested it as a crucial test." P. 137.

A description of several cases follows confirmatory of the statement contained above; and we need only add, that we have heard from another source of high authority of the disappearance of phosphatic deposits under alkaline treatment, the remedy exhibited being in this case at first bicarbonate of lime in solution, and afterwards lime water.

Dr. Rees does not propose this treatment as universally effectual, but affirms his certainty, that in the greater number of cases in which alkaline and neutral urine exists with phosphates as a deposit, we shall find alkaline remedies of avail. He gives reasons for the opinion that the phosphates in these cases are precipitated from the urine as the result of the neutralizing action of an alkaline mucus on its acids, but not secreted by the mucous membrane, and he thinks it of importance to distinguish the cases where the phosphates, though precipitated, do not exceed the normal amount passed in the urine from those in which their amount is preternaturally increased; the last condition, he conceives, attends some important constitutional diseases, as for instance mollites ossium in children. In the view taken of the sources of oxalate of lime deposits, the number of vegetables in common use containing oxalic acid, or the oxalates, as more especially the stalks and roots of rhubarb, and the leaves of French sorrel, are not sufficiently adverted to; the experiments of Dr. Aldridge are referred to, wherein it is shown that boiling alone produced crystals of oxalate of lime in healthy urine, and it is presumed that the acid may be formed from the decomposition of lithic acid; it is to be remembered, however, that the fluids cannot be boiled within the body, and we have no indication of any action within the system which can have an equivalent power. Upon how many theories of this kind may we pronounce the verdict of—not proven. In cases of this kind, as in numerous others, we fall back upon the plan approved by experience of improving, as far as we can, the functions of the digestive organs, and skin, with little guidance derived from the existence of this particular deposit, and, alas! but little assistance derived from mere chemical sources.

We fully assent to the proposition that, as far as treatment is concerned, little advancement has been made with regard to albuminuria; it is in its advanced stages a most formidable and intractable disease. Recent investigations, we are told, have shown the structural alteration of the kidney to be of the nature of a fatty degeneration; but, alas! how little practical knowledge do we at present obtain by assimilating it with an analogous change in the liver.

We demur, on the authority of Dr. Christison, to the statement that, in every stage of granular disease of the kidney, we observe the urine to contain albumen. We believe that in an advanced stage albumen may be altogether absent, though liable to re-appear from trifling causes; indeed this is subsequently admitted by Dr. Rees himself. Our author's experience is valuable in confirmation of the generally-received opinion, that the solid matter evacuated with the urine during the day in the later as well as early stages of this affection, is far below the proportion of health; he mentions the fibrin in the blood as generally exceeding the natural amount, the albumen as often much diminished, the specific gravity of the serum, which in health is about 1029, having been once found by him as low as 1015; the deficiency of albumen he speaks of as attendant upon the early, that of hæmatosine upon the later stages of the disease. Our own experience has long ago satisfied us of the ready susceptibility of those labouring under this disease to the action of mercury, but we have never seen any injurious consequences from its use, and Dr. Rees seems to concur in both these conclusions. In the description of the course and complications of this disease we find nothing that has not been stated in the full and accurate treatise of Dr. Christison, unless it be the liability to pericardiac inflammation.

As regards treatment, we believe, with the last named author, that anasarca dependent on albuminuria *alone*, is not an untractable form of disease: we fancy that we have often warded off apoplexy by watching constantly, and acting promptly, whenever distinct threatenings were discerned, and on such occasions we have not hesitated to bleed. But for the vomiting, sometimes alone, sometimes alternating with diarrhœa, we have in some cases found no remedy and little relief, and for this state of things we have no suggestion from our author. The treatment of the acute stages with diaphoretics, and of the less active symptoms with iron, is, we conceive, judicious: opium we eschew or give cautiously, lest it mask the inroads of head affection.

Our space will not allow us to enter at any length upon the remarks made on the subject of Diabetes: the observation that, even in advanced cases the sugar on some days is not to be detected in the urine, while the high specific gravity of the secretion is kept up by the presence of an enormous excess of urea is very curious, and bears a strong analogy to Dr. Prout's observation of the saccharine condition being at times preceded by great abundance of urea. The use of opium, we think, is here too disparagingly spoken of, we have treated few but very far advanced cases: in one, in which we gradually carried its exhibition to the extent of six grains three times a day, the general symptoms were decidedly improved, though the secretion of sugar was little if at all diminished; no inconvenience arose from the administration of these doses, which neither constipated the bowels nor produced drowsiness. Iron is recommended by Dr. Rees, and we think favourably of some of its preparations, which we have tried. One paragraph on the diet seems well deserving attention. As regards the diet best suited to this form of disease, it appears anything but reasonable to subject the stomach to the severe discipline which has been applied by the fashion of the day in prohibiting the use of vegetable food, and restricting the patient to a purely animal diet." The exhibition of magnesia is recommended, but whether this treatment is dictated by theory or experience, we are not told. Warm bathing at first, and cold sponging and cold bathing when they can be borne, are also favourably spoken of.

It will be seen, from what has just preceded, that the views of Dr. Rees on the subject of treatment are independent and sometimes original, founded on his own reasonings and experience, rather than taken up as matter of fashion or caprice. There is one thing that we especially admire in this part of his work, namely, that though so good a chemist he has no disposition to make the experiments of the laboratory overrule the lessons to be learned only in the sick-room; he takes into account the influences of a vital action as well as the affinities of chemistry.

